# *In vivo* and *in vitro* ageing results in accumulation of *de novo* copy number variations in bulls

**DOI:** 10.1038/s41598-017-01793-2

**Published:** 2017-05-09

**Authors:** Tamas Revay, Olutobi Oluwole, Tom Kroetsch, W. Allan King

**Affiliations:** 1 Department of Biomedical Sciences, University of Guelph, Guelph, ON Canada; 2 Semex, 5653 HWY6 North, Guelph, ON Canada

## Abstract

We have identified *de novo* copy number variations (CNVs) generated in bulls as they age. Blood samples from eight bulls were collected and SNP arrayed in a prospective design over 30 months allowing us to differentiate *de novo* CNVs from constant CNVs that are present throughout the sampling period. Quite remarkably, the total number of CNVs doubled over the 30-month period, as we observed an almost equal number of *de novo* and constant CNVs (107 and 111, respectively, i.e. 49% and 51%). Twice as many *de novo* CNVs emerged during the second half of the sampling schedule as in the first part. It suggests a dynamic generation of *de novo* CNVs in the bovine genome that becomes more frequent as the age of the animal progresses. In a second experiment *de novo* CNVs were detected through *in vitro* ageing of bovine fibroblasts by sampling passage #5, #15 and #25. *De novo* CNVs also became more frequent, but the proportion of them was only ~25% of the total number of CNVs (21 out of 85). Temporal generation of *de novo* CNVs resulted in increasing genome coverage. Genes and quantitative trait loci overlapping *de novo* CNVs were further investigated for ageing related functions.

## Introduction

Copy number variants (CNVs) are deleted or duplicated segments of the genome that have been identified as a prominent source of inter-individual genetic variation. A current CNV map estimates that 4.8–9.5% of the human genome can be affected by these events without apparent phenotypic consequences^1^. CNVs however alter the balance of DNA content and have been associated with disease conditions, such as diabetes, autism and schizophrenia^2^. Recently the CNV landscape of many other species including domestic animals has also been investigated^3^. The potential to affect gene expression or regulation defines CNVs as candidate selection markers for modern breeding schemes; e.g. milk yield in cattle and fertility in pigs have been associated with various CNVs^4, 5^.

The mechanisms of DNA replication and repair are not only essential for the divisions producing the trillions of cells of a whole organism, but are also major factors in generating CNVs and genetic diversity^6^. The resulting intra-individual somatic variability has been observed at the level of CNVs among differentiated tissues of humans and cattle^7, 8^. The progressive and systematic changes of cellular structures are hallmarks of ageing, thus the temporal generation of CNVs is a logical subject of longevity or ageing related studies. On the other hand, the dynamics of *de novo* somatic CNV formation is an understudied aspect of the biology of ageing. Kuningas *et al*.^9^ have observed two common CNV regions and the burden of large common CNVs were associated with higher mortality among the participants of the Rotterdam and Farmingham human ageing studies. Similarly, the increasing total length of genome-wide CNVs were associated with mortality in another population study^10^. Accumulation of CNVs in human blood and colon cells over time was also described^11, 12^. Although cattle CNVs have been investigated in a number of studies, most are oriented to reveal variability in populations and potential associations with production traits^13^ and temporal changes of the CNVs landscape has never been described.

The aim of our study was to investigate the dynamics of CNV formation *in vivo* and *in vitro* during prospective sampling of individual bulls and fibroblast cultures. Our aim was not to create a snapshot from the genomes of very mature individuals, as in many genetic studies of ageing, instead to carry out an experiment in bulls where the sequential sampling provides follow up information on the same individual genomes. These *in vivo* and *in vitro* models of the changing CNV landscape provide novel information of the extent of *de novo* CNVs in bulls as well as their potential connection to ageing related genomic changes.

## Results

### CNV analysis

The experiments aimed to detect and analyze CNVs from data collected on the Bovine SNP50 k chip were performed on two sets of samples. First, the *in vivo* ‘AGE’ array consisted of testing DNA from blood samples of eight bulls collected at three consecutive time points (at 14 ± 3month of age, then 18 months and 30 months later, Supplementary Figure S1). Moreover, three bovine fibroblast cell lines were established and DNA samples extracted from three consecutive passages (P5, P15, P25) tested on the *in vitro* ‘FIBRO’ array.

Several quality control steps were performed for both arrays prior to CNV scoring and analysis. Analysing samples by either the derivative log ratio data or the genomic waves characteristic yielded no outlier samples. Although the 24 DNA samples from the eight bulls (AGE) were optimally processed together since they completely filled one array (24 positions), we investigated potential batch effects by principal component analysis (Supplementary Figure S2). This revealed no clustering of the raw data according to the sampling time and minimal tendency of grouping based on the genetic origin (animals). All nine fibroblast samples were arrayed together on the FIBRO array as well. The PCA did not detect clusters by cell passages, but similarity of samples from the same cell lines were observed.

The Univariate-CNAM algorithm of the SVS software detected CNVs by identifying segments of logR ratio values that are significantly different from the neighbouring values. This procedure searches along the genome in individual samples, generating a list of segments that were then critically evaluated (using the segment mean histogram and visualization of the genomic loci) to form a final list of 218 CNVs in total from the *in vivo* and 85 CNVs from the *in vitro* samples. That makes a very similar average number of CNVs/sample for the two dataset (27 CNVs/AGE samples and 28 CNVs/FIBRO samples). The majority of CNVs were deletions, as only eight and seven gains were detected *in vivo* and *in vitro*, respectively, as also observed in earlier studies using the same platform^14^. The mean and median length of a CNV was slightly, but not significantly, longer for AGE than for FIBRO (mean: 61 kb vs. 47 kb, median: 31.9 kb vs. 31.7 kb, respectively). The individual CNVs were named and numbered according to the dataset, as CNV-A1 to CNV-A218 for AGE and CNV-F1 to CNV-F85 for FIBRO (Tables S2, S3 lists all CNVs).

**Figure 1:**
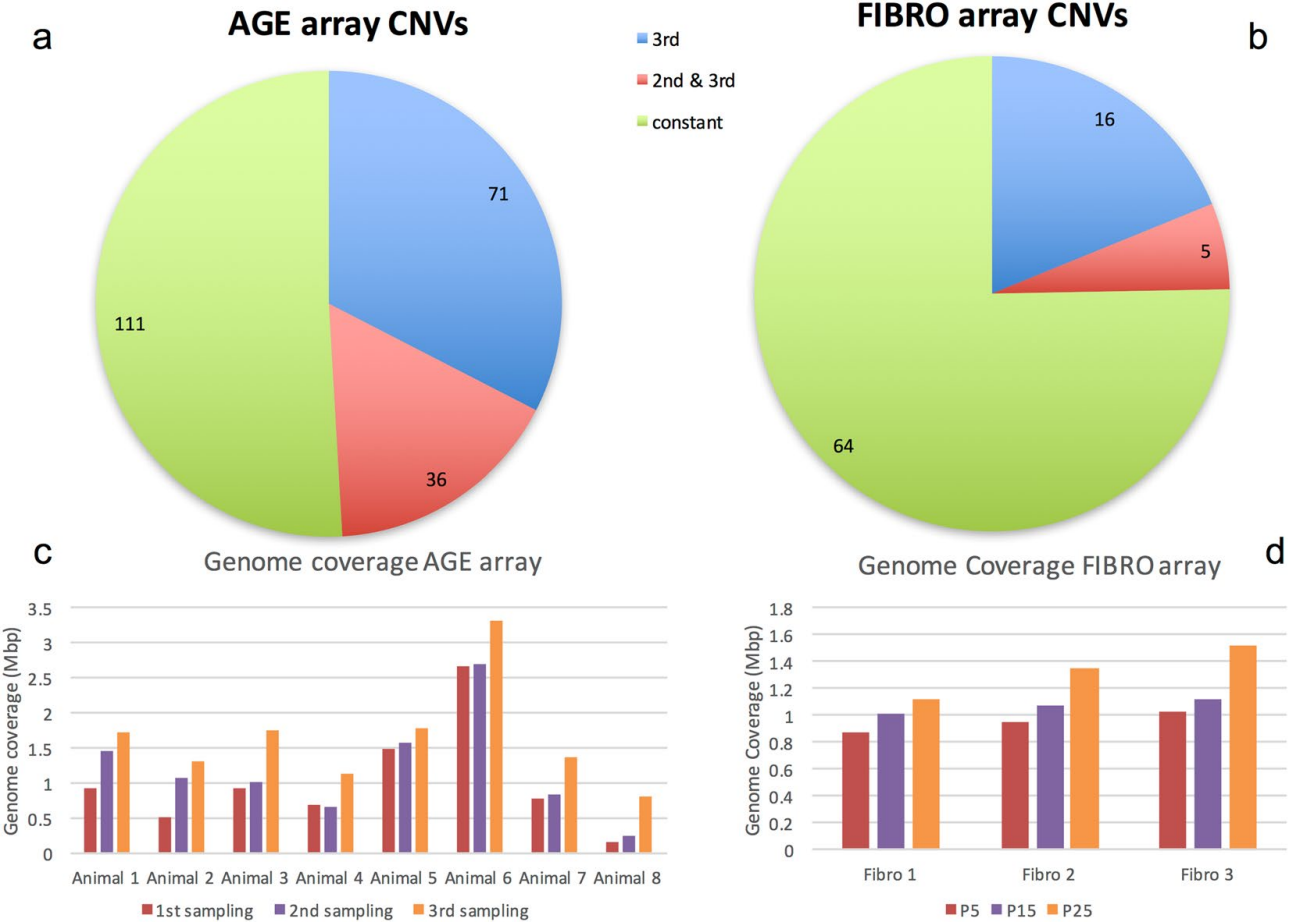
Pie charts presenting the total number of CNVs identified as “constant”, “2nd & 3rd” time point or “3rd” sampling. These latter two classes were scored as “*de novo*”. The ratio of *de novo* vs. constant CNVs was significantly different between AGE (**a**) and FIBRO (**b**) (p < 0.0001). Genome coverage of AGE array samples (**c**) and FIBRO array samples (**d**) at the three time points. Cumulative increase of the portion of genome involved in CNVs are visible as *de novo* CNVs added to the constant CNVs over time.

The next analysis step was to group and compare CNV calls among the three consecutive samples from the same bulls or cell lines (Table S1). CNVs detected at all three time points represent events that exist in the genome since the beginning of the sampling scheme e.g. a deletion that was present in the genome of one particular bull at the age of 12 months and detected again at later times in the 2nd and 3rd sample collected from the same animal. Similarly, there are CNVs that could be found in all passage samples of a particular fibroblast line. These events are referred to as “constant”, reflecting the fact that they did not change over the sampling period (Table S1). We found 111 constant CNVs *in vivo* and 64 *in vitro*. On the other hand, we looked for CNV events that were not present in the first sample, but arose later in the sampling schedule. If a CNV was detected in the 2nd sample (18 months later or P15) a scoring criterion was its presence in the 3rd time point as well. There were 36 *in vivo* and five *in vitro* CNVs detected in the 2nd and 3rd set of samples but not in the 1^st^ set. Moreover, there were 71 *in vivo* and 16 *in vitro* CNVs of the latter class, those detected only in the 3rd sample (30 month later or P25). Due to the fact that these last two categories of CNVs were not present at the beginning of the experiments, but presumably generated *de novo* during the time the sampling was scheduled we refer to them as “*de novo*”. There were 107 *in vivo* and 21 *in vitro de novo* CNVs. The ratios of total *de novo* vs. total constant CNVs for both *in vivo* and *in vitro* samples were significantly different (p < 0.0001, binomial test), as were the ratios of average *de novo* vs. average constant CNVs per sample (Fig. 1a,b). The genome coverage was calculated for each sample and reflected an increasing length when *de novo* CNVs were added at the 2^nd^ and 3^rd^ time points to the portion covered originally by the constant CNVs (Fig. 1c,d).

No constant or *de novo* CNV was detected on BTA26 (for AGE) or on BTA13, BTA18, BTA20 for FIBRO (Supplementary Figure S4). The largest number of CNVs were detected on BTA2, BTA4, BTA8 and BTA12 for AGE and on BTA2, BTA3, BTA11 for FIBRO, while eight other chromosomes had only one CNV. Individual counts of either constant or *de novo* (2nd & 3rd or 3rd) CNVs varied among the bulls or cell cultures (Supplementary Figure S5). Constant CNVs were in excess in most cases, especially for the *in vitro* samples. However, the number of merged *de novo* events resulted in inverting this difference in six out of eight *in vivo* samples, while it did not change the surplus of constant CNVs in all *in vitro* samples (Supplementary Figure S5c,d). There were on average 14 constant and 13 *de novo* CNVs per *in vivo* sample (median = 14 for both CNV classes), and 21 constant and 7 *de novo* CNVs per cell line (median = 22 and 7, respectively).

A quantitative PCR (qPCR) validation of 28 *de novo* CNVs (20 *in vivo* and 8 *in vitro*) were performed and the results were in 80% agreement with the array-based status predictions (Table S4).

### CNV gene content

**Figure 2:**
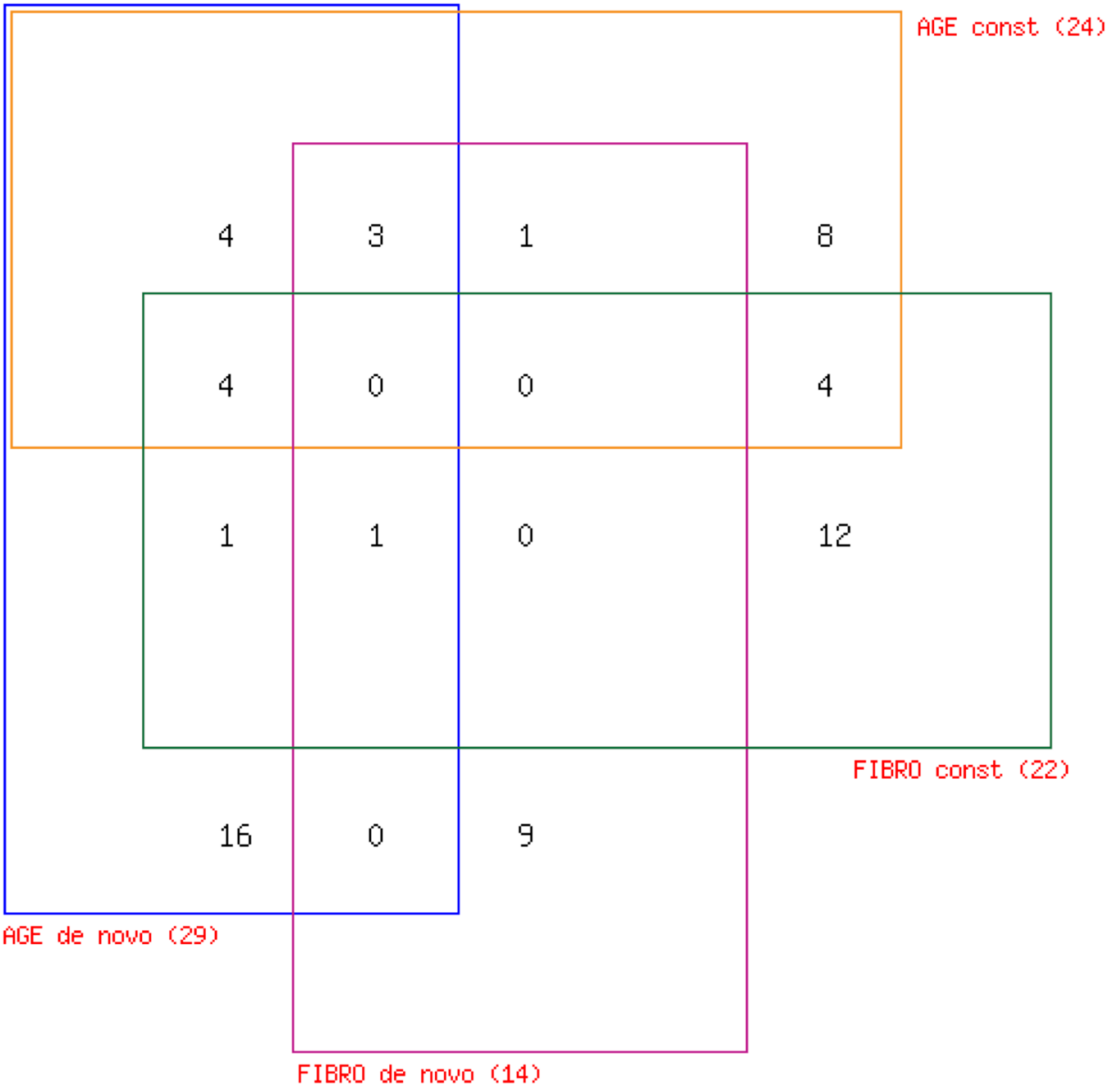
Venn diagram representing genes shared by or unique to AGE and FIBRO CNVs.

Positions of all identified CNVs were used to search for overlapping bovine RefSeq genes. 35 AGE CNVs and 26 FIBRO CNVs encompassed a total of 63 genes with 25 unique to *de novo* CNVs (Fig. 2). The highest number of genes (16) were associated with *de novo* AGE CNVs and nine to *de novo* FIBRO CNVs, as listed in Table 1. We found three molecular functions, including “unfolded protein binding”, “GTPase regulator activity”, “nucleoside-triphosphatase regulator activity” enriched significantly among the genes overlapping FIBRO *de novo* CNVs. The observed common biological functions for AGE *de novo* gene set were “protein complex subunit organization”, “macromolecular complex assembly” or “regulation of cellular ketone metabolic process”, although they were not significantly enriched (Table 2).

**Table 1:** List of genes overlapping genomic positions with *de novo* AGE or FIBRO array CNVs.

AGE *de novo*	FIBRO *de novo*
SLC25A24	CELF4
BOLA-DRB3	DAXX
ACN9	ELMOD1
ARHGAP26	MIR2376
B3GALNT2	PFDN6
C2CD5	RGL2
CERS6	SLC25A21
DOCK10	TAPBP
DZIP3	WDR46
EIF2AK3	
FANCC	
KCND3	
KIAA1524	
MATN2	
SLAMF8	
SLC39A11

**Table 2:** Functional enrichment analysis of genes overlapping *de novo* CNVs.

Array	Molecular function	GO ID	Statistics	adjusted P-value
FIBRO	unfolded protein binding	0051082	C = 127; O = 2; E = 0.06; R = 34.38; rawP = 0.0014	adjP = 0.0112
	GTPase regulator activity	0030695	C = 446; O = 2; E = 0.20; R = 9.79; rawP = 0.0162	adjP = 0.0453
	nucleoside-triphosphatase regulator activity	0060589	C = 458; O = 2; E = 0.21; R = 9.53; rawP = 0.0170	adjP = 0.0453
AGE	regulation of cellular ketone metabolic process	0010565	C = 168; O = 2; E = 0.11; R = 17.42; rawP = 0.0056	adjP = 0.2743
	macromolecular complex assembly	0065003	C = 995; O = 3; E = 0.68; R = 4.41; rawP = 0.0262	adjP = 0.3065
	protein complex subunit organization	0071822	C = 894; O = 3; E = 0.61; R = 4.91; rawP = 0.0197	adjP = 0.2743

Where C = number of reference genes in the category, O = observed number of genes in the gene set from the category, E = expected number in the category, R = Ratio of enrichment, rawP = p value from hypergeometric test, adjusted p-value = p value adjusted by the multiple test adjustment.

### CNVs overlapping QTL regions

**Figure 3:**
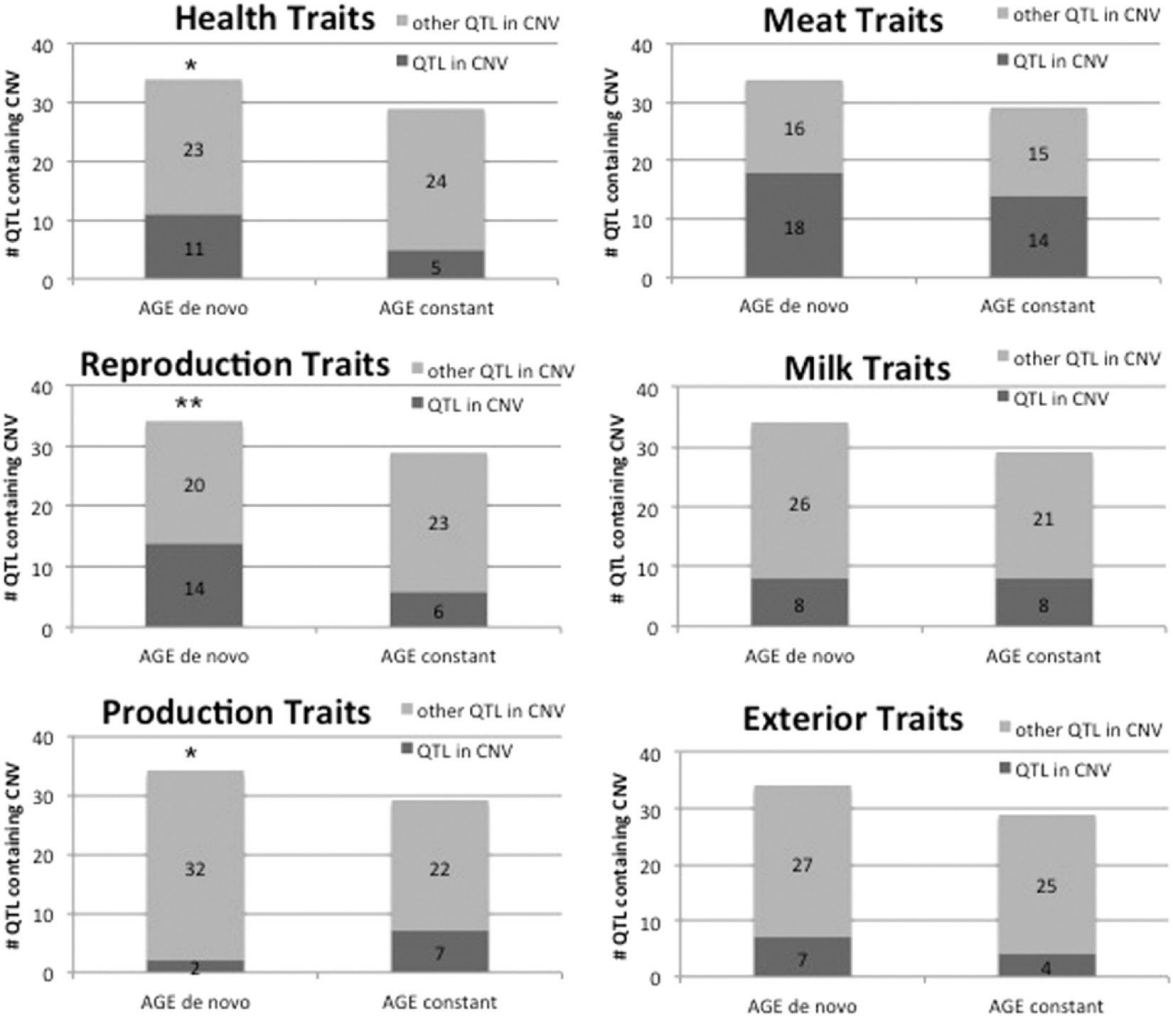
Number of major QTL classes assigned to the *in vivo* AGE CNVs. *p < 0.05, **p < 0.01.

**Figure 4:**
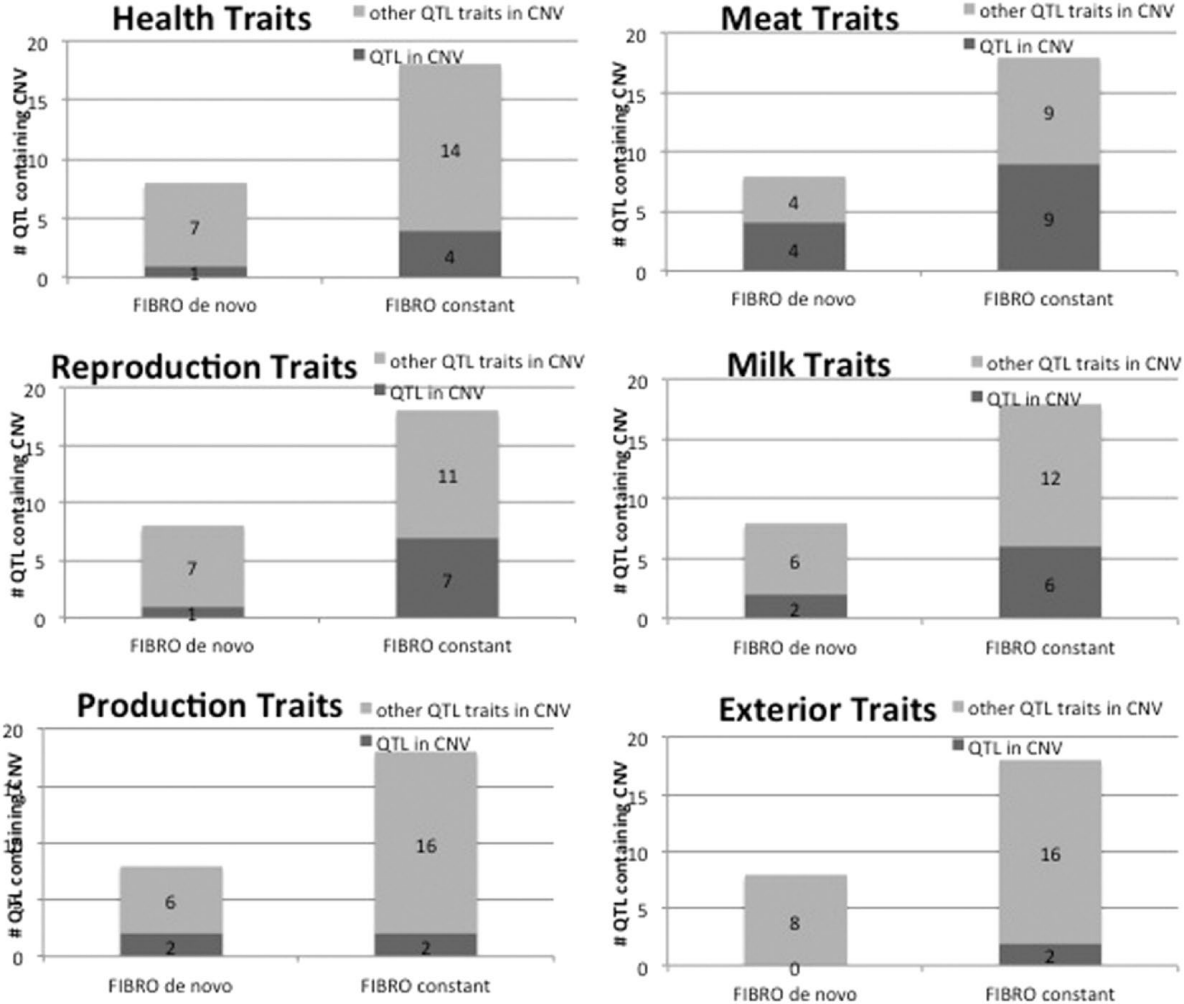
Number of major QTL classes assigned to the *in vitro* FIBRO CNVs.

The genomic positions of the identified CNVs were compared to that of mapped bovine QTLs. Approximately one third of the CNVs overlapped with QTL regions (Supplementary Figure S6). The average number of QTLs falling into a CNV region varied between 8 and 17, the most observed in *de novo* AGE CNVs (Supplementary Figure S6). Each CNV locus was annotated by the six major QTL classes (Health, Meat and carcass, Milk, Production, Reproduction, Exterior traits) mapped to the position of the CNV. The most frequent QTL classes were Meat and carcass, Reproduction and Health traits, e.g. out of 34 *de novo* AGE CNVs that encompassed any class of QTLs, 18 overlapped with Meat QTLs. Significant enrichment of Health (p = 0.036, binomial test) and Reproduction QTLs (p = 0.009, binomial test) were observed in *de novo* vs. constant *in vivo* CNVs, while no significant enrichment of Production trait QTLs was found (Fig. 3). No statistical enrichment was found in any QTL classes between *de novo* and constant *in vitro* CNVs (Fig. 4).

## Discussion

In this study, we have identified *de novo* CNVs generated in bulls as they age. The samples were collected in a prospective design allowing us to differentiate *de novo* generated CNVs from constant CNVs that were present throughout the sampling period. This is the first time that individual bulls were sequentially sampled - three samples collected over 30 months - in order to detect *de novo* CNVs that were further characterized for genomic signatures of ageing. Quite remarkably, the total number of *in vivo* CNVs doubled over the 30-month period, as we observed an almost equal number of *de novo* and constant CNVs (107 and 111, respectively, i.e. 49% and 51%, Fig. 1a). Interestingly, the median size of constant and *de novo* CNVs were not different from each other (32 kb and 36 kb). Thus, the total genome coverage (calculated by multiplying the median CNV length by the number of CNVs) was also not different between constant and *de novo* CNVs (3.5 Mbp vs. 3.9 Mbp, respectively). Investigating the 2^nd^ and 3^rd^ sampling time points, it was noted that twice as many *de novo* CNVs emerged during the second half of the sampling schedule as in the first part (a total of 71 and 36, respectively, i.e. on average 9 and 5 per animal, Fig. 1a). It suggests a dynamic generation of *de novo* CNVs in the bovine genome that becomes more frequent as the age of the animal progresses. This results in the temporal cumulative increase in genome coverage in all samples (Fig. 1c), as the *de novo* CNVs were generated and added to the constant CNV fraction. This is in agreement with the observation of a burden of CNVs in human population studies that compare young vs elder subjects^9, 10^, however our study design was able to reveal the activity of de novo CNV formation, as an explanation for this observation. Hsieh *et al*.^11^ have also found accumulation of structural chromosomal changes in human colon crypts.

Consistently, the *in vitro* FIBRO array also showed an increasing number of *de novo* CNVs over the tested consecutive passages of bovine fibroblast cell lines (a total of five at the 2^nd^ sampling vs. 16 at the 3^rd^, Fig. 1b). However, the proportion of *de novo* CNVs detected in fibroblasts was only ~25% of the total number of CNVs (21 *de novo* vs. 64 constant CNVs, Fig. 1b). This is a highly significant difference, as compared to the 49% *de novo* CNVs *in vivo* (p < 0.0001, binomial test). This could be due to the different characteristics of the two experimental models, that are *in vivo* blood-forming cells over 30 months vs. *in vitro* fibroblasts cultures over 3–5 months of passages. CNVs are generated by various mechanisms (recombination, DNA repair) during the cell cycle^6^ and the number of cell divisions that occurred during the two experiments could have been different. We can estimate ~50 divisions *in vitro* between P5–P25 with an average of 2.5 population doublings per passage, compared to a minimum of 120 divisions *in vivo* considering less than a week renewal time for lymphocytes^15, 16^. The difference of the rate of *de novo* CNV generation remains even after being corrected by the estimated number of divisions (107/120 = 0.9 *in vivo* versus 21/50 = 0.4 *in vitro*). However, additional factors, such as the impact of the individual genetics, the unknown effects of culture conditions or *in vivo* stress, etc. cannot be excluded. The median size of constant and *de novo* CNVs were not significantly different from each other (33 kb and 30 kb, respectively), however the smaller number of FIBRO samples added up to lower total genome coverage than the AGE samples (2.2 Mbp constant and 630 kbp *de novo*). Nonetheless, we consider these two experiments as complementary to each other providing information on both *in vivo* and *in vitro* dynamics of *de novo* CNV formation in the bovine genome.

CNVs in the cattle genome have been detected in a number of studies designed to explore population diversity or to identify associations with production traits^13^. Although none of these studies have differentiated between *de novo* and constant CNVs, the general properties of CNVs analysed using the SNP array technology are comparable to the ones identified here. The median length of AGE and FIBRO CNVs was very close to each other (~32 kb), that is on the shorter end of the 21 kb–5.6 Mb size range published for cattle^14, 17, 18^. This variability of CNV calls was mostly caused by the difference of the algorithms used to segment the raw data, as compared by Xu *et al*.^14^, but the local variability of probe density could also play a role. We have chosen the SVS software based on our previous experience^4^ and the rich available options for quality control and visualization.

Functional characterization of *de novo* CNVs was attempted by locating genes overlapping *de novo* CNV loci. We found that nine genes map to the region covered by the 21 *in vitro* CNVs, while only 16 genes are encompassed by the 107 *in vivo* CNVs. This could be interpreted as 3x higher chance for a *de novo* CNV in fibroblast to be generated in coding region - thus potentially affect gene function - than it is *in vivo* in blood cells, although the smaller *in vitro* sample size could have affected this comparison.

The functional characteristic of genes overlapping *de novo* CNVs (both AGE and FIBRO) were further investigated using the various tools embedded in the Webgestalt software package as well as the comprehensive collection of ageing related changes of the GenAge database. Gene ontology analysis revealed functions connected to proteostasis, that is one of the hallmarks of not only fibroblast ageing, but also the general ageing related alterations of cellular functions^19, 20^. Furthermore, ARHGAP26 – a gene overlapping an AGE CNV -, connects to “GTPase regulator activity” and the GenAge member ARHGAP1. This latter gene is implicated in the regulation of cytoskeleton, affecting cellular adhesion and motility that are linked to premature ageing^21^ and age-related diseases^22^. Several SLC genes were found in *de novo* CNVs (SLC25A21, SLC25A24, SLC39A11) that are involved in body-fat composition and distribution in human and mice and found to be associated with ageing in a large Korean cohort study^23^. Interestingly another member of this gene family (SLC6A14) was also affected by a CNV in humans^24^. Another gene overlapping with an AGE CNV is ceramide synthase 6 (CERS6), which is related to mitochondrial dysfunction by acting as a pro-apoptotic factor^25^. Another pro-apoptotic protein DAXX which overlapped with a FIBRO CNV, has been identified in Drosophila to affect longevity^26^, and also acts as a target of the REST transcription factor, that protects against Alzheimer’s and normal ageing^27^.

Another advantage of our bovine model for characterization of *de novo* CNVs is the availability of a detailed map of genomic regions associated with variation of quantitative traits (QTL map). The analysis of overlapping QTL classes provided an additional layer of functional characterization to the CNVs. Surprisingly, health and reproduction traits are over-represented in *de novo* AGE CNVs. In contrast, milk traits – that give ¼ of all 42000 cattle QTLs and production traits QTLs, such as feed intake or daily gain, are under-represented in *de novo* CNVs. On the other hand, previous research into bovine CNVs found associations with milk production or residual feed intake in diverse selected populations of cattle^5, 18^.

The current study identified *de novo* CNVs in ageing bulls and bovine fibroblast cultures. The highly dynamic generation of these CNV events as well as their potential links to ageing-related genomic functions were observed and thus warrant further studies of larger populations and the application of higher-resolution arrays or genome sequencing, which will improve the sensitivity of CNV detection.

## Methods

### Animal samples for AGE array

Peripheral blood samples were collected from eight Holstein bulls housed and managed under the same conditions at the same Canadian farm. Sampling was done according to the Canadian Council on Animal Care (CCAC, http://www.ccac.ca/en_/standards/policies) and the University of Guelph’s Animal Care Committee (ACC, http://www.uoguelph.ca/research/services-divisions/animal-care-services) guidelines by licensed farm technicians or CFIA (Canadian Food Inspection Agency) veterinarians. The animals were not selected for research purposes, blood samples left over from regular health check events were used for DNA extraction. The animals were available for three consecutive sampling over the course of almost three years. The bulls were approximately one year old at the start of the sampling. The collection time points (14 ± 3month, 18months later: 32 ± 3 m, 12months later: 44 ± 3 m) are shown in Figure S6.

Genomic DNA was extracted from the blood samples using a traditional Proteinase K digestion, salting out and phenol/chloroform method^28^ and concentrations were determined by a NanoDrop photometer. The three samples from the eight animals (total of 24) were submitted to a genomic service provider (DNA LandMarks, QC, Canada) where they were genotyped on the Illumina Bovine SNP50k array. The 24 samples were handled on the same day, processed on one array slide according to the laboratory SOP. This dataset was referred throughout the manuscript as the AGE or *in vivo* dataset.

### Fibroblast cultures

Primary fibroblast cell lines were established by using standard culture techniques^29^ from ear notch samples of three unrelated Holstein bulls slaughtered at a local abattoir. These animals were not related to the ones genotyped on the AGE array. Briefly, the samples were digested in 0.5% collagenase type 1 for 6 h at 38 °C then cultured in DMEM with 10% FBS, 1% PenStrep (Invitrogen Canada Inc. Burlington, ON, Canada) and 2 mM L-glutamine (Sigma-Aldrich Canada Ltd, Oakville, ON, Canada). The primary fibroblasts were sequentially cultured (seeded at 3 × 10^4^ cells/25 cm^2^ flask and harvested at ~1.5 × 10^5^ cells/flask, ~2.5 population doubling/passage) and sampled at passages 5, 15 and 25 (P5, P15, P25) for DNA extraction.

DNA samples were genotyped on the Illumina Bovine SNP50k array at the same facility (DNA LandMarks), as the AGE array according to the same protocol. The nine samples (3 × 3) were processed on one array slide on the same day, although it was a different day than for the AGE array. This dataset was named FIBRO or *in vitro* dataset.

### CNV analysis

The raw data, that is the log R ratio, represents a normalized signal intensity value for each probe on the array, thus it can be related to the amount of DNA hybridized to over 54,000 loci along the bovine genome^30^. The data discussed in this publication have been deposited in NCBI’s Gene Expression Omnibus^31^, and are accessible through GEO Series accession number GSE95612 (https://www.ncbi.nlm.nih.gov/geo/query/acc.cgi?acc=GSE95612).

The log R ratio values were calculated using the Illumina GenomeStudio software, before transferring the data to the SNP and Variation Suite (SVS version 8.4.3., GoldenHelix Inc.) for CNV analysis. First, several quality control steps were taken to identify low quality samples. Noise in the log R ratio values generally comes from the sample preparation or genotyping procedures and could be identified by testing for outliers in the median derivative log ratio values. Genomic waves that represent the variation of log R values according to the DNA composition targeted by the probes were detected by Diskin *et al*.^32^. The X and Y chromosomal data were excluded from the data. Potential batch effects were tested by principal component analysis (PCA) of logR values. SNP annotations were according to the representative genome Bos_taurus_UMD_3.1.1 (2014).

CNVs were identified in SVS by the Univariate CNAM algorithm that scans log R values of each sample separately and identifies DNA segments that are significantly different from their neighbors. The segmentation conditions were set to 0.005 maximum pairwise segment p value, ≥2 markers/segment with outlier removal function and the segment means were filtered to be <−0.35 for losses or >0.35 for gains. The resulting list of CNVs (named as CNV-A for AGE and CNV-F for FIBRO array) was classified according to their presence in consecutive samples from the same animal or cell culture. CNVs present in all three-samples corresponding to the three time points of sampling (blood samples collected at different ages of the same bull or passages P5, P15 and P25 of the same cell line) were named as “constant”. Alternatively, CNVs detected in the 2nd and 3rd or only at the 3rd sampling time were classified as “*de novo*”. The ratio of *de novo* vs constant CNVs were compared between AGE and FIBRO with the binomial test, while the CNV length were compared using the Kruskal-Wallis test in Prism 6 (GraphPad). CNVs that could not be scored either as “constant” nor “*de novo*” were inconsistent to our model and not considered further. These types of CNVs (listed in Table S6) were the ones detected only at the 1^st^ or the 2^nd^ time point or the 1^st^ and 2^nd^ time points and probably were caused by changes of the abundance of cells carrying the variant or its mosaic status reaching below detection limit. No CNVs were identified at 1^st^ and 3^rd^ time points, showing consistency of detection.

qPCR tests were designed to validate 28 different 3^rd^ time point *de novo* loci including all samples. The tests were run in triplicate across all three time points and a control sample. This control sample should function as a reference, stably representing diploid copy number for all of the tested CNV loci and was created as a mixture of all 1^st^ time point samples. qPCRs were run in a CFX96 Touch™ Real-Time PCR Detection System (Bio-Rad) using a thermal profile of 98 °C, 2 min; 45 × (98 °C, 10 sec; 59 °C, 10 sec), followed by the registration of a melting curve between 68 °C to 95 °C in 0.5 °C/sec increments. The 10 µl reaction was composed of 1 × SsoFast EvaGreen Supermix (Bio-Rad), 3 mM primers and 20 ng genomic DNA. Relative quantity was calculated in the Bio-Rad CFX Manager software against the control sample and two normalizer genes SRY and BTF3, both widely used for qPCR validation of bovine CNVs. Then the ratio of 2^nd^ and 3^rd^ time point data to the 1^st^ was calculated to reveal copy number decrease or increase. The PCR efficiency was calculated using the actual amplification curves using the LinReg PCR software^33^ Primers were designed using the NCBI Primer-BLAST tool, except for BTF3^34^ (Table S5).

### Functional annotation of CNVs

Genomic locations of all bovine QTLs were downloaded from the Animal Genome Database (release 28^35^). Enrichment of a particular trait category in *de novo* vs. constant CNVs was detected by the binomial test.

The RefSeq genes were downloaded by using the UCSC Table browser^36^. The resulting list of annotated genes for each array classes (AGE, FIBRO or *de novo*, constant) were compared by generating a four-way Venn diagram using the web-tool developed by Chris Seidel and available from http://www.pangloss.com/seidel/Protocols/venn4.cgi.

Both *de novo* gene lists (AGE and FIBRO) were further analyzed against the GenAge database (http://genomics.senescence.info/genes/index.html ^37^) and for functional enrichment in Gene Ontology (GO) terms, using the WEB-based Gene SeT AnaLysis Toolkit (WebGestalt)^38^. The bovine gene names were converted to the corresponding human ones and the human genome was used as reference set for the default statistical test (Benjamini-Hochberg, adjusted p-value < 0.01).

## Electronic supplementary material

Supplementary Information

Supplementary table S1-S6
