# Supplementary material for: In vivo and in vitro ageing results in accumulation of de novo copy number variations in bulls
Source: Sci Rep. 2017 May 9;7:1631. doi: 10.1038/s41598-017-01793-2 (PMC5431667; doi:10.1038/s41598-017-01793-2)

***In vivo* and *in vitro* ageing results in accumulation of de novo copy number variations in bulls**

Tamas Revay, Olutobi Oluwole, Tom Kroetsch, W. Allan King

Supporting Information

Figure S1. The age of bulls at the three sampling time points.

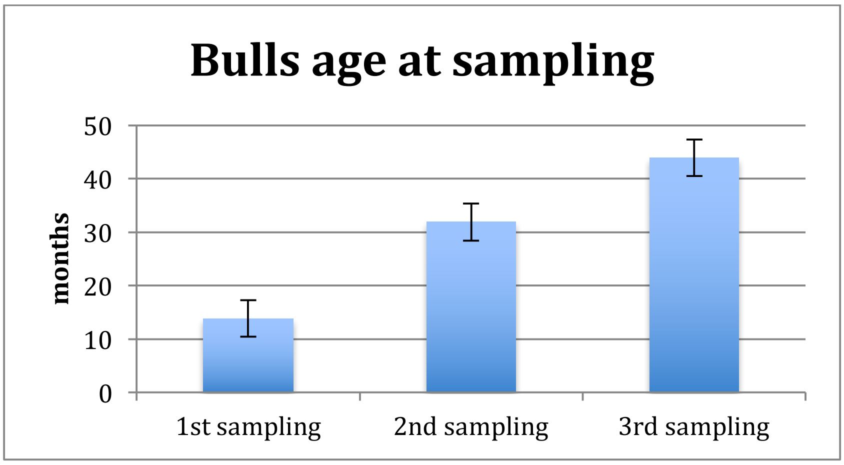

Figure S2. Principal component analysis (PCA) of the AGE (A, B) and FIBRO (C,D) array samples. The two largest principal components were used as X- and Y-axis. Samples are color coded according to the sampling time point (A: age of bulls, C:cell passage) or genetic origin (B:bull, D: cell line). On A: blue, green and orange marks correspond to time point #1, #2 and #3, respectively. On B: animals are coded as 101= Animal 1, 110= Animal 2, 111= Animal 3, 113= Animal 4, 114= Animal 5, 117= Animal 6, 119= Animal 7, and 128= Animal 8.

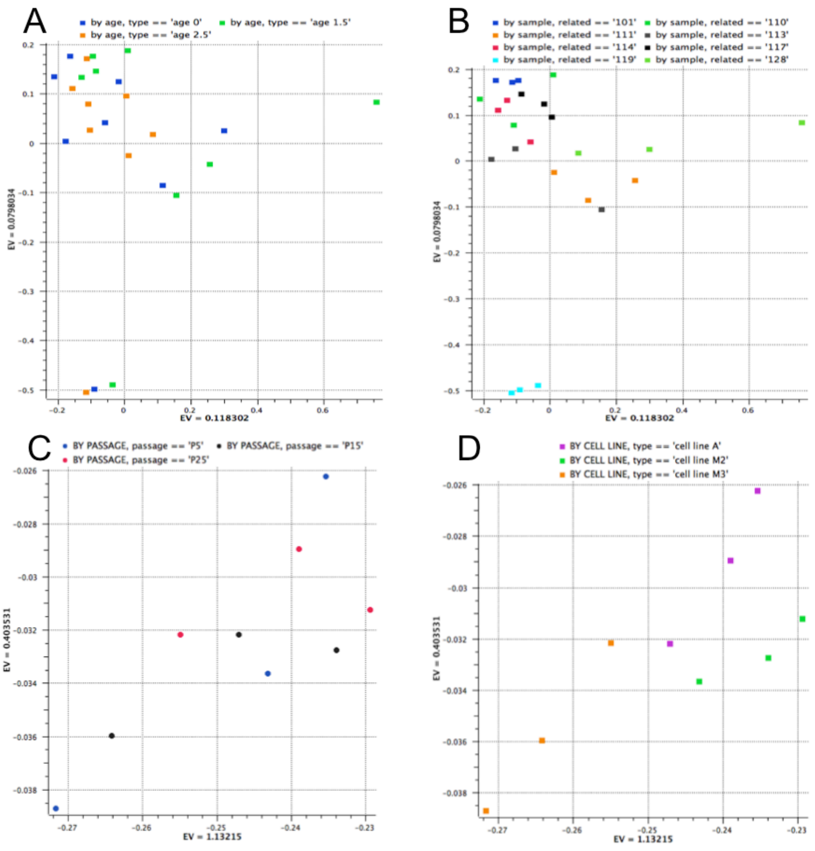

Figure S3. Representative view of a segment of the bovine genome. The distribution of logR ratio and BAF values are shown for three samples (sample #28 three time points). The deviation of LR from '0' and the BAF values from '1 or 0.5' led to the detection of a deletion in all three samples. The heat map below identifies this constant deletion CNV as vertical red bars across the samples where present. E.g. all three time point from sample 28 contain a deletion. The brown rectangles on the CNV scoring track mark the positions and help visualization. The green-blue track at the bottom of the figure is the positions of Ensemble RefSeq genes. Animals are coded as 1.1= Animal 1 – time point 1, 1.4=Animal 1 – time point 2, 1.6=Animal 1 – time point 3, accordingly, 10= Animal 2, 11= Animal 3, 13= Animal 4, 14= Animal 5, 17= Animal 6, 19= Animal 7, and 28= Animal 8.

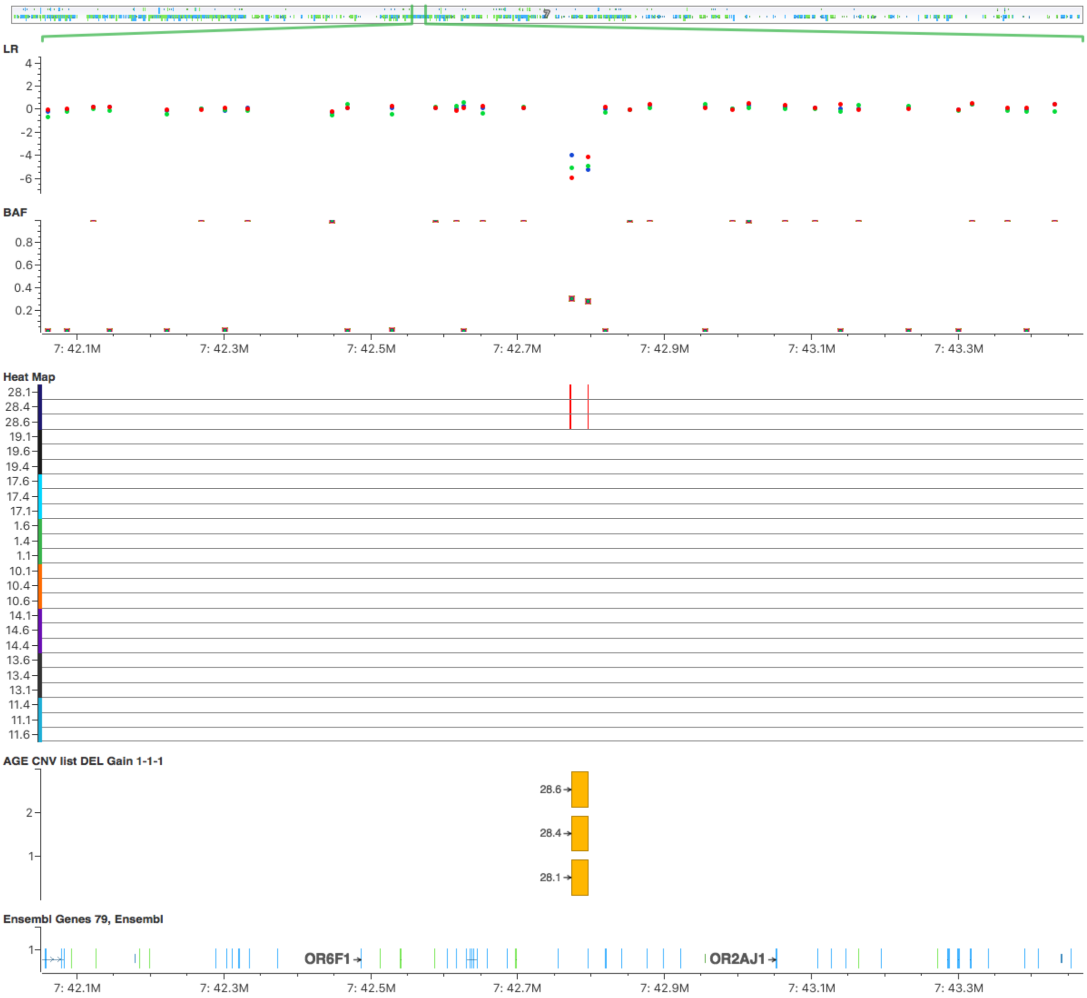

Figure S4. The distributions of CNVs on individual bovine chromosomes.

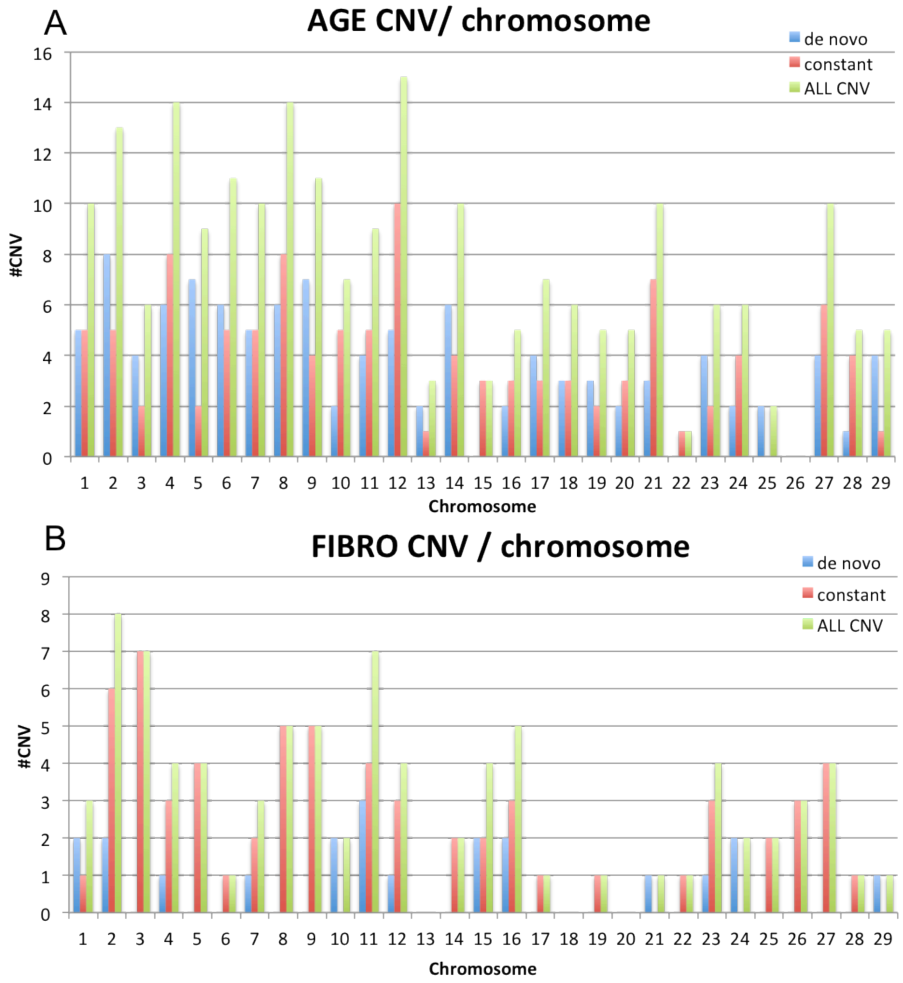

Figure S5. The number of individual CNVs in each class of events detected. A, B present all three classes of constant, 2nd &3rd, 3rd only CNVs, while C, D contrast de novo CNVs with constant CNVs.

Animals are coded as 1=Animal 1, 10= Animal 2, 11= Animal 3, 13= Animal 4, 14= Animal 5, 17= Animal 6, 19= Animal 7, and 28= Animal 8. Fibroblasts coded as A=A, B=M2, C=M3.

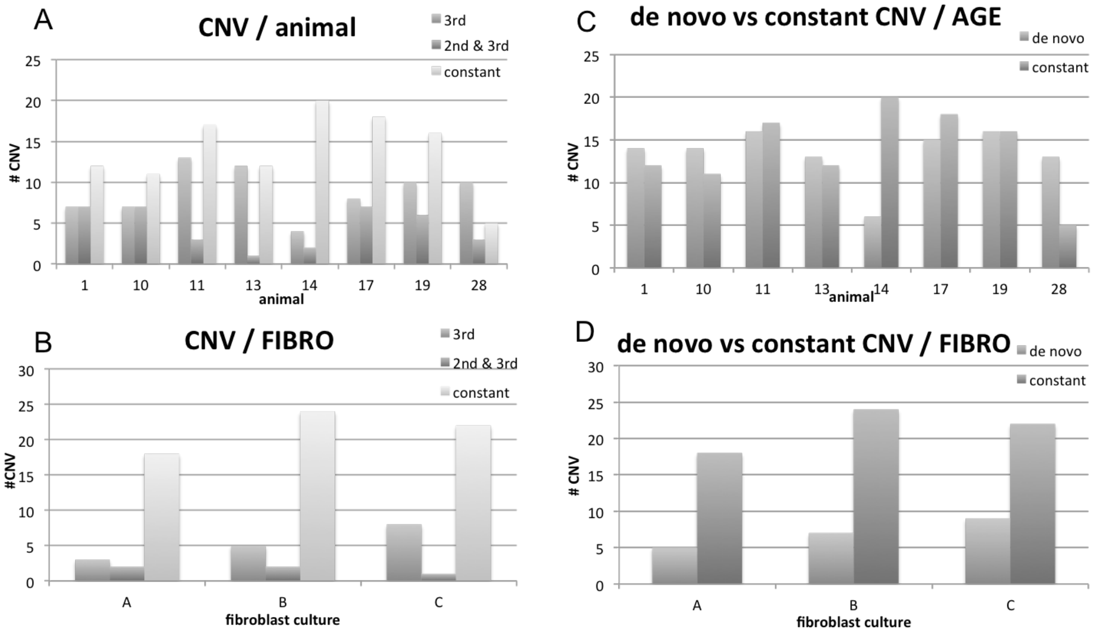

Figure S6. Numerical analysis of CNVs overlapping QTLs.

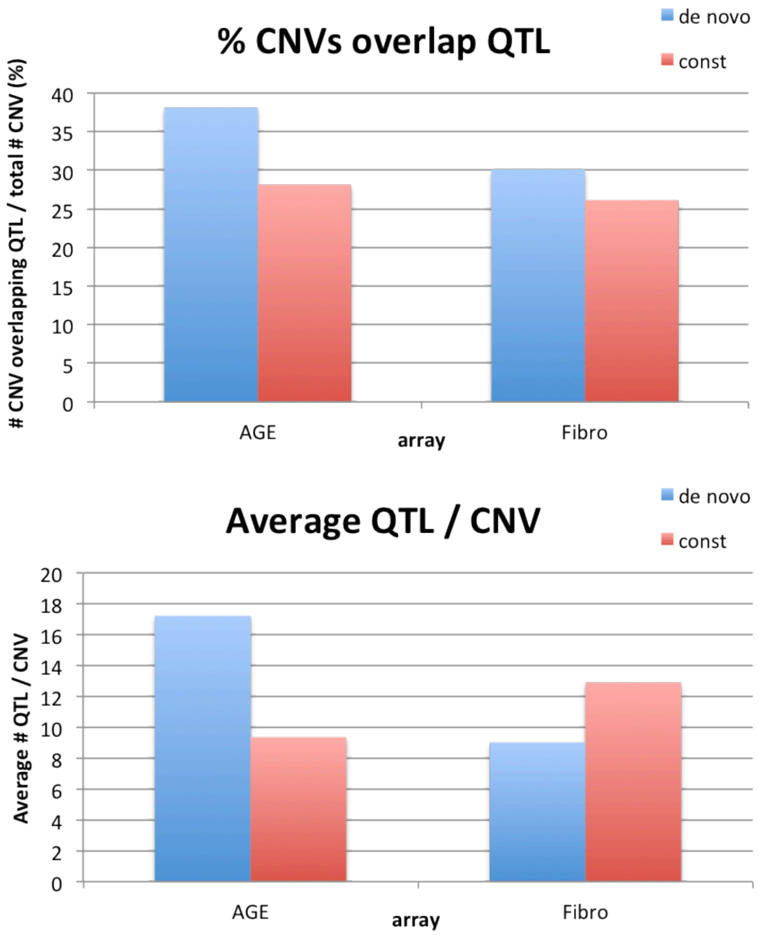

Supplement: Supplementary file 1 — Supplementary Information [file 41598_2017_1793_MOESM1_ESM.pdf]
